# Asymmetric metabolic adaptations undermine stability in microbial syntrophy

**DOI:** 10.1093/ismeco/ycaf011

**Published:** 2025-01-25

**Authors:** Nan Ye, Zhi-Chun Yang, Zhuang-Dong Bai

**Affiliations:** School of Ecology and Environment, Northwestern Polytechnical University, Xi’an 710129, China; Shaanxi Key Laboratory of Qinling Ecological Intelligent Monitoring and Protection, School of Ecology and Environment, Northwestern Polytechnical University, Xi’an 710129, China; School of Ecology and Environment, Northwestern Polytechnical University, Xi’an 710129, China; Shaanxi Key Laboratory of Qinling Ecological Intelligent Monitoring and Protection, School of Ecology and Environment, Northwestern Polytechnical University, Xi’an 710129, China; School of Ecology and Environment, Northwestern Polytechnical University, Xi’an 710129, China; Shaanxi Key Laboratory of Qinling Ecological Intelligent Monitoring and Protection, School of Ecology and Environment, Northwestern Polytechnical University, Xi’an 710129, China

**Keywords:** syntrophy, metabolite production, metabolite utilization, population dynamics

## Abstract

Syntrophic interaction, driven by metabolite exchange, is widespread within microbial communities. However, co-inoculation of most auxotrophic microorganisms often fails to establish a stable metabolite exchange relationship. Here, we engineered two auxotrophic *Escherichia coli* strains, each dependent on the other for essential amino acid production, to investigate the dynamics of syntrophic relationships. Through invasion-from-rare experiments, we observed the rapid formation of syntrophic consortia stabilized by frequency-dependent selection, converging to a 2:1 ratio of lysine-to-arginine auxotrophs. However, laboratory evolution over 25 days revealed that syntrophic interactions were evolutionarily unstable, with cocultures collapsing as ΔL cells dominated the population. Reduced fitness in cocultures was driven by the emergence of a “selfish” ΔL phenotype, characterized by decreased arginine production and exploitation of lysine produced by ΔA cells. Dynamic metabolic assays revealed that metabolite production and utilization patterns strongly influenced the fitness of each strain. ΔL cells displayed metabolic plasticity, adjusting lysine utilization in response to lysine availability, which enabled them to outcompete ΔA cells. In contrast, ΔA cells lacked similar plasticity, resulting in their negative selection. These findings demonstrate that asymmetric metabolic responses and the emergence of selfish phenotypes destabilize syntrophic relationships. Our work underscores the importance of balanced metabolic exchanges for developing sustainable synthetic microbial consortia and offers insights into the evolutionary dynamics of microbial cooperation.

## Introduction

Microbial communities play a pivotal role in the function of most ecosystems, such as contributing to the viability of plants and animals [1], as well as fostering global biogeochemical cycling [2]. A prevalent interaction within microbial communities is syntrophy, defined as the cooperative exchange of metabolites between two or more organisms where at least one partner cannot complete a metabolic process without the assistance of the other [3]. The metabolite exchange within syntrophic consortia influences the composition and stability of microbial communities [4, 5]. Despite the importance of microbial communities, the factors that determine their stability or how a community responds to biotic and/or abiotic perturbations are poorly understood. This limits our broader understanding of the ecological functions that microorganisms fulfill.

To elucidate the fundamental biological and ecological dynamics of microbial interactions, engineered consortia have been established, predicated on the partitioning of metabolic functions among diverse microorganisms [6–8]. In the laboratory, various types of auxotrophic microbial strains have been synthesized through targeted metabolic pathway engineering [9]. However, there are divergences regarding the capability of auxotrophic strains in developing sustainable cross-feeding syntrophic relationships. For instance, auxotrophs with complementary amino acid and nucleotide metabolisms often fail to compensate for each other’s deficiencies unless they are genetically modified to increase metabolite exports [7]. Conversely, certain auxotrophic strains have been observed to spontaneously establish stable syntrophy [9–11]. These seemingly contradictory findings raise an urgent question: What factors underlie the challenges faced by some auxotrophic strains in achieving stable metabolic interchange with other strains within a microbial consortium?

One potential challenge to establishing nutritional interdependence within microbial communities is that co-growing auxotrophic cells produce metabolites to benefit themselves, but the excess exported metabolites are insufficient to fully support the growth of a complementary partner [7, 12]. Several strategies that may enhance metabolic cooperation among auxotrophs have been explored, including facilitating their interaction within spatially structured environments to minimize metabolite dissipation [13, 14] or removing any feedback inhibitory effects on metabolite production, thereby converting the target bacteria into metabolite overproducers [7, 15]. Although these measures aimed at improving the efficiency of metabolite exchange contribute to the stability of engineering synthetic communities, they are not essential for communal equilibrium. Microorganisms preferentially utilize metabolites excreted by other cells over their own [16]. Hence, auxotrophic cells possess the potential for the exchange of metabolites essential for growth. It has been demonstrated that auxotrophic strains can spontaneously form mutually beneficial interactions with other auxotrophic strains in the absence of extensive genetic manipulation and optimization [9, 16–18]. Therefore, the lack of a syntrophic relationship among auxotrophs cannot be attributed solely to insufficient production or export capacity. Indeed, increased metabolite production does not necessarily promote syntrophy, as the auxotrophs’ metabolite utilization also affects the interaction outcome [19]. We posit that, for certain auxotrophic strains, specific metabolite utilization patterns may pose challenges to the establishment of a sustainable interaction.

To elucidate the mechanisms that may destabilize bacterial syntrophic interactions, we engineered two auxotrophic strains of *Escherichia coli* (*E. coli*), each dependent on the essential amino acids produced by the other for sustainable growth. The stability or immediate collapse of the syntrophy between different strains depends on the production and consumption of metabolites [20]. Hence, we examined fluctuations in metabolite production and utilization in a cross-feeding consortium continuously cultured for 25 days. We observed that in one strain metabolite utilization correlates with the metabolite production from the other strain, reflecting the adaptation of this auxotrophic strain to its localized environment. However, the two auxotrophic strains differed in their capacity to respond to the environment, resulting in the faster-responding auxotroph increasing its own fitness at the expense of the other. Our results show that asymmetries in metabolic function between two interdependent *E. coli* strains can result in the destabilization of syntrophic interactions.

## Materials and methods

### Strains and culture conditions

We used *E. coli* MG1655 (CCTCC AB209131) from the China Center for Type Culture Collection as the wild-type (WT) strain. Two knockout mutant strains were generated using CRISPR-Cas9 genome editing technology [21]. Briefly, a Cas9 plasmid was transformed into WT cells, followed by the preparation of WT-Cas9 competent cells. The target sequence for sgRNA binding to specific genes was designed online (https://zlab.bio/guide-design-resources) and subsequently amplified by polymerase chain reaction (PCR) to bind to the sgRNA plasmid. Donor DNA fragments, containing 500 bp homologous sequences flanking the target gene, were synthesized by fusion PCR and co-electroporated with the sgRNA plasmid into WT-Cas9 competent cells, generating auxotrophic mutants [21]. The resulting mutants either lacked the *lysA* (ΔL) or *argH* (ΔA) genes, encoding enzymes for the terminal biosynthesis steps of lysine (Lys) or arginine (Arg), respectively, thus conferring distinct metabolic capabilities. The mutants were sequenced to confirm that the *lysA* or *argH* deletions were the sole mutations, with no off-target effects. Sequencing details are delineated in the Supplementary methods. To distinguish the strains phenotypically, we transformed plasmids GSH_KTNR_GFP or GSH_KTNR_mCherry (Fig. S1), which constitutively express green (GFP) or red fluorescent protein (mCherry) and confer kanamycin resistance, into the two auxotrophic genotypes. Kanamycin (50 μg/ml) was used to maintain the fluorescent markers during culture. These two plasmids carried fluorescent proteins (GFP and mCherry) showed very limited effects on cell growth and biomass accumulation (Fig. S2). Comprehensive information on plasmid and primers can be found in the supplementary information (Tables S1 and S2).

After CRISPR-Cas 9 modification and plasmids transformation, samples from both modified cultures were collected and stored at −80°C for later experimental analysis. In this study, all cultures were incubated at 37°C whilst shaking at 220 rpm in M9 medium (6.8 g/L Na2HPO4, 3 g/L KH2PO4, 1 g/L NH4Cl, 0.5 g/L NaCl, 2 mM MgSO_4_·7H_2_O, 0.5 mM thiamine hydrochloride, 0.0025 g/L FeSO_4_·7H_2_O and 0.5% (wt/vol) glucose) (Table S3). Auxotrophic strains were pre-cultured in M9 medium supplemented with 150 μM lysine or arginine, harvested at the late-exponential phase, and washed three times in M9 medium by centrifugation at 12 000 rpm for 5 minutes. Cell densities were determined by optical density readings (Biotek Synergy Neo2, Vermont, America) at 600 nm (OD_600_) and validated by colony-forming units per milliliter (CFUs/ml) on selective M9 agar plate supplemented with lysine or arginine. Precultures were diluted to OD_600_ ~ 0.1 and inoculated into fresh M9 medium at a 1:100 volume ratio to standardize initial conditions across replicates for subsequent experiments.

### Phenotypic verification of auxotrophy

We confirmed the auxotrophic phenotype of the engineered *E. coli* by pre-culturing six colonies of each auxotrophic strain under the conditions described above. Each colony was then inoculated into three distinct media conditions: (i) M9 medium supplemented with 150 μM of lysine or arginine (positive control), (ii) M9 medium without amino acid supplementation (negative control), and (iii) M9 medium supplemented with 150 μM of all other proteinogenic amino acids (control for potential biochemical conversion to the focal amino acid). We incubated the cultures at 37°C with shaking (220 rpm) for 24 hours and measured OD_600_ using optical density readings (Biotek Synergy Neo2, Vermont, America) at 600 nm (OD_600_) to assess growth.

### Invasion-from-rare experiments

The syntrophic stability and coexistence dynamics of the two auxotrophic strains were assessed using reciprocal invasion-from-rare experiments. For this, each auxotrophic strain was co-inoculated into M9 medium at initial frequencies of 1:100 and 1:10 relative to its partner strain. As a control, the two strains were co-inoculated at an initial 1:1 ratio. A total initial cell density of ~2 × 10^5^ cells/ml was introduced into M9 medium without amino acid supplementation. Cultures were incubated at 37°C with shaking (220 rpm). The relative ratio of the two strains was quantified by calculating the ratio of CFUs/ml of ΔL to ΔA at 0, 48, 72, and 96 hours post-inoculation. Each experimental condition was performed in four independent replicates.

### Laboratory evolution

This experiment involved three groups: Group 1 contained a monoculture of the prototrophic WT strain cultivated in M9 medium without amino acid supplementation. Group 2 included monocultures of the lysine or arginine auxotrophs (ΔL and ΔA), each grown in M9 medium supplemented with a sufficient concentration (150 μM) of the respective amino acid. Group 3 comprised cocultures of ΔL and ΔA in unsupplemented M9 medium, inoculated at an initial 1:1 ratio. Each group was replicated eight times under identical experimental conditions, except for medium composition.

Ancestral strains were retrieved from refrigerated storage, defrosted, and re-activated on Lysogeny Broth (LB) agar plates containing kanamycin (50 μg/ml). Eight colonies of each strain were picked, pre-cultured in M9 medium supplemented with 150 μM of the relevant amino acid and kanamycin, and grown at 37°C whilst shaking (220 rpm) for 12 hours. These pre-cultures were subsequently washed, diluted to OD_600_ ~ 0.1, and used to inoculate both monocultures and the coculture. The same ancestral colonies served as the starting material for all monoculture and coculture experiments.

For monocultures, 25 μl of each pre-culture dilution was inoculated into 5 ml of M9 medium with the respective amino acid (150 μM) and incubated at 37°C with shaking (220 rpm) for 24 hours. Daily, 50 μl of culture was transferred into a fresh medium under identical conditions. Cocultures were initiated by inoculating 25 μl of ΔL and ΔA pre-cultures into 5 ml of M9 medium without amino acid supplementation. Cocultures were incubated for 5 days before transferring 50 μl into a fresh medium, repeating the cycle until one of the cocultures ceased growth. This procedure spanned 25 days (five transfers), equating to ~35 generations. The initial CFUs/ml density of ΔL (1.01 ± 0.18) × 10^5^ and ΔA (0.94 ± 0.17) × 10^5^ confirmed comparable starting conditions. Optical densities (OD_600_) were measured by microplate spectrophotometry (Thermo Fisher Scientific), and CFUs / ml were determined at each transfer cycle. The experimental duration was based on monoculture and coculture growth curves (Fig. S2) and previous research on auxotrophic *E. coli* evolution [10]. At the end of each transfer cycle, eight derived colonies from each replicate were isolated and preserved in 15% glycerol at −80°C for downstream analyses. Fitness changes were quantified by comparing the biomass accumulation of evolved strains relative to their ancestral counterparts.

### Relative fitness of ancestral versus evolved population

To evaluate evolutionary changes, the relative fitness of evolved populations (Evo) was compared to their corresponding ancestral populations (Anc). Day 25 isolates from WT, monoculture, and coculture conditions, along with their respective Day 0 counterparts, were pre-cultured as described above. Pre-cultures were then inoculated into fresh 5 ml M9 medium with or without amino acid supplementation and incubated for 24 hours (WT, monocultures) or 72 hours (cocultures). CFUs were measured by plating on M9 agar at 0 and 24 or 72 hours. Relative fitness was calculated as:


$$ \mathrm{Relative}\ \mathrm{fitness}=\ln\ \left({\mathrm{N}}_{\mathrm{t},\mathrm{Evo}}/{\mathrm{N}}_{0,\mathrm{Evo}}\right)/\ln\ \left({\mathrm{N}}_{\mathrm{t},\mathrm{Anc}}/{\mathrm{N}}_{0,\mathrm{Anc}}\right) $$

where N_0_ is the initial CFUs count and N_t_ is the final CFUs count. Each evolutionary lineage was assessed with eight biological replicates.

### Competitive fitness assays

Competition experiments were conducted by co-inoculating the competing strains at equal densities (~10^5^ cells/ml) into the specified test media. Frequencies of the competing strains were determined at 0 and 24 hours by plating on M9 agar with or without amino acid supplementation. To quantify fitness relative to the WT, each auxotroph (ΔL or ΔA) was competed against WT in M9 medium supplemented with the respective amino acid. Additionally, cocultures of the two auxotrophs (ΔL + ΔA) were competed against WT to assess the fitness of the binary consortium relative to WT. For the three-strain competitions, ΔL, ΔA, and WT were inoculated at an initial ratio of 0.5:0.5:1 in M9 medium supplemented with both lysine and arginine, maintaining a total cell density of ~2 × 10^5^ cells/ml. This experiment was conducted in four independent replicates. To evaluate fitness changes before and after the laboratory evolution experiment, auxotrophic strains from three different conditions were tested: (i) ancestral strains from Day 0, (ii) auxotrophs evolved in monoculture by Day 25, and (iii) auxotrophs evolved in coculture by Day 25. Each condition was compared against the WT to quantify the fitness changes resulting from evolutionary adaptation.

### Relative fitness of strains isolated from cocultures

The relative fitness of derived ΔL and ΔA strains from cocultures was evaluated using the Malthusian parameter, which quantifies population growth rate per unit time [22]. Briefly, 5 μl of overnight culture (OD_600_ ~ 1.0), prepared from a single derived or ancestral auxotrophic colony, was inoculated to 5 ml M9 medium supplemented with relevant amino acids (150 μM). CFUs/ml were measured at 0 h and 24 hours of incubation at 37°C with shaking (220 rpm). The realized biomass accumulation over 24 h of the derived or ancestral ΔL and ΔA (the Malthusian parameter M) was calculated as:


$$ M=\frac{\ln \left(\frac{N_t}{N_0}\right)}{t} $$

where *N_0_* is the initial population density at 0 h, *N_t_* is the final population density after 24 h [22] and t is 24 h. The relative fitness was determined by dividing the M value of each derived strain by the M value of its corresponding ancestor. Eight biological replicates were performed for each cycle of the experiment. The 24-hour incubation period allowed ΔL and ΔA strains to transition from the logarithmic phase to the stationary phase, capturing comprehensive growth dynamics. To quantify evolutionary changes, the relative fitness approach compared the growth of derived strains to the same ancestral counterparts, standardizing the analysis across strains with different transfer histories and accounting for any consistent effects of stationary-phase growth. Representative growth curves for both auxotrophic strains are provided in supplementary information (Fig. S2), illustrating the dynamics under these experimental conditions.

### Confocal laser scanning microscopy

Confocal laser scanning microscopy (CLSM) (Nikon AX Confocal Microscopes, Tokyo, Japan) equipped with a 20× objective was employed to quantify the frequencies of ΔL and ΔA strains within coculture replicates. Coculture samples were diluted ~1000-fold in M9 medium, and 5 μl of the dilution was transferred onto microscope slides. CLSM was then used to count mCherry and GFP cells within an 880 μm × 880 μm visual field. Fluorescence signals were captured using a 561 nm laser line (606–695 nm emission window) for mCherry and a 488 nm laser line (535–580 nm emission window) for GFP, respectively. To mitigate potential spatial variability of bacterial distribution on slides, three to six technical replicates were performed for each cocultured biological replicate.

Captured images were analyzed with ImageJ v.1.53 s software (https://imagej.nih.gov/ij/, Rasband, 1997–2022) [23]. The procedure consisted of four steps: (I) scale calibration (menu “Set Scale”), (II) transforming the fluorescent image from RGB color to 16-bit (menu “Type”), (III) color masking (menu “Color Threshold…”), and (IV) cell counting (menu “Analyze Particles” with size (pixel^2) from 5 to infinity) [24]. In the resulting tables, cell counts (Count) were shown. To validate the accuracy of subpopulation percentages derived from CLSM, these data were compared with the CFU ratios (ΔL relative to ΔA) obtained from the same coculture samples using selective plating (“Strains and culture conditions”). No significant differences were observed between the two methods for determining the relative abundances of ΔL and ΔA within cocultures (Fig. S3).

### Metabolite utilization

The auxotrophic strains isolated from monocultures and cocultures were pre-cultured in M9 medium supplemented with 150 μM lysine or arginine at 37°C, 220 rpm conditions for 12 hours. Precultures were washed in amino acid-free media and subjected to starvation at 37°C for 4 ~ 6 h to deplete intracellular lysine or arginine reserves. Starved cells (2 μl OD_600_ ~ 0.1) were inoculated into 198 μl M9 medium containing varying concentrations of lysine (0, 0.5, 1, 2, 4, 8, 16 μM) or arginine (0, 0.5, 1, 2, 4, 8, 16, 32 μM) [25]. These concentrations were chosen to ensure a coefficient of determination (R^2^ > 0.95) in the linear regression between metabolite concentration and cell density, based on the method of Bertels et al. (2012). Cultures were incubated at 37°C with shaking at 220 rpm for 24 hours, after which cell densities (CFUs/ml) were measured. We performed a linear regression between input metabolite concentrations (explanatory variable) and final cell densities (response variable) within the linear range, forcing the regression line through the origin (intercept set to zero). Metabolite utilization per cell in a saturated culture was quantified as the reciprocal of the regression slope (1/slope) (Fig. S4) [19]. Eight biological replicates were analyzed for each auxotrophic strain from the cocultures and monocultures at every cycle of the laboratory evolution experiment.

### Comparison of utilization

Isolated ΔL strain with lysine utilization capacities of 2 fmole/cell and 10 fomle/cell were streaked on freshly prepared LB agar plates. Colonies differing in lysine utilization were selected and grown overnight in M9 medium supplemented with 150 μM lysine at 37°C with shaking (220 rpm). Overnight cultures were diluted to OD_600_ ~ 0.1, and 2 μl of each dilution was inoculated into 198 μl M9 medium supplemented with 50 μM lysine in a 96-well plate. Cultures were incubated for 24 hours at 37°C with shaking at 880 rpm. OD_600_ was recorded in a plate reader (Biotek Synergy Neo2, Vermont, America) every hour. The intrinsic growth rate was determined from growth curves using the logistic equation fit calculated using the “Growthcurver” package in R [26]. This experiment was replicated four times.

### Metabolite production level assay

To quantify lysine or arginine production by ancestral and derived strains isolated from cocultures and monocultures during the laboratory evolution experiment, these strains were used as amino acid donors in co-incubation assays with auxotrophic biosensor *E. coli* strains [25]. Ancestral ΔL and ΔA strains stored at −80°C were revived to act as biosensors. Biosensor growth, which depends on the amino acids provided by the donor strains, was used as an integrative measure of donor amino acid production [10, 27]. Donor and biosensor colonies were pre-cultured in M9 medium supplemented with lysine or arginine (150 μM) until the exponential phase. Pre-cultures were washed in an amino acid-free medium and diluted to the OD_600_ ~ 0.1. Equal volumes (25 μl each) of diluted donor and biosensor cultures were inoculated into 5 ml M9 medium and incubated at 37°C, 220 rpm for 72 hours. Identical biosensors were used to compare amino acid production levels across donor strains from different treatments and evolution cycles.

After 72 hours, the relative growth of the biosensor population compared to the donor was determined using CLSM. The relative biosensor growth level—representing the number of biosensor cells supported per donor cell—was calculated as: Relative biosensor growth = N_b_ / N_d_, where N_b_ is the number of biosensor cells and N_d_ is the number of donor cells. The amino acid production level by the donor population was calculated as: Amino acid production level by the donor population = N_b_ / N_d_ × CFU_d_, where CFU_d_ is the donor population density in laboratory evolution experiment. This assay was replicated eight times for each biosensor–donor combination. To verify that donor-produced amino acids were fully utilized by the biosensors, 1 ml of supernatant from each coculture was extracted and supplemented with an equal volume of sterile-filtered 2 × M9 medium. Subsequently, 10 μl of biosensor preculture (OD_600_ ~ 0.1) was added to the supernatant and incubated for 24 hours. No detectable increase in optical density was observed in these cultures, confirming that all donor-produced amino acids had been exhausted during the co-incubation period.

### Quantitative analysis of gene expression

Quantitative PCR (qPCR) was employed to monitor the changes in the expression level of key genes involved in lysine (*lysA*, *dapA*) [28] and arginine (*argA*, *argH*) [29] biosynthesis. These measurements were used to infer the trends in amino acid synthesis of auxotrophic strains under different transfer intervals. Detailed protocols for RNA extraction, cDNA synthesis, and qPCR are provided in the supplementary methods and figures (Fig. S8).

### Statistical analyses

The normality of the error variances of all measures were assessed using Shapiro-Wilks and Levene’s tests. Parametric tests were applied when their underlying assumptions were met. For comparisons between two independent groups, two-tailed independent t tests were used, while paired sample t tests were used for paired comparisons between two non-independent groups. Differences among non-independent groups across multiple time intervals were assessed using one-way repeated measures ANOVA followed by a Bonferroni post hoc test. For comparisons among three or more independent groups, one-way ANOVA followed by a Bonferroni post hoc test was applied. When parametric assumptions were violated, non-parametric tests were applied. The Wilcoxon signed-rank test was used for two non-independent groups, while the Friedman rank test for three or more non-independent groups. One-sample t test was conducted to determine whether the relative fitness of derived auxotrophs significantly deviated from 1, which represents ancestral or WT fitness. Pearson’s product–moment correlation was used to test the relationship between two variables. *P*-values for multiple comparisons among non-independent groups were adjusted by applying the Bonferroni correction [30]. Statistical analyses were performed using SPSS 25.0 (SPSS Inc., Chicago, IL, USA) for general statistical tests, and the “Growthcurver” (growth rate assay) and “dplyr” (correlation assay) packages in R 4.3.2 (https://cran.r-project.org/) for specialized analyses.

## Results

**1. Auxotrophs can form syntrophic consortium in a short time** 

**Fig. 1:**
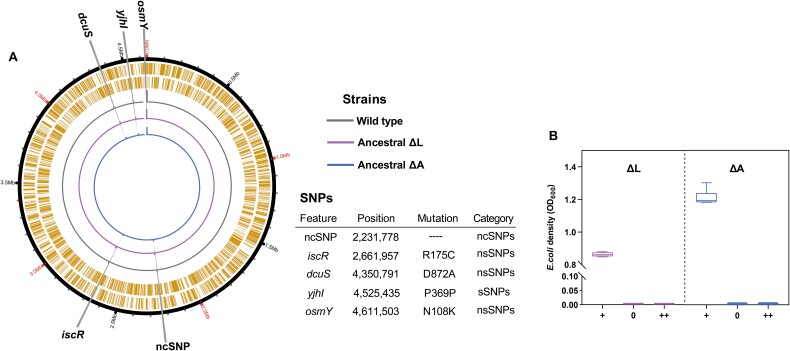
**Confirmation of the phenotypic specificity of auxotrophs.** (A) Whole-genome sequencing of various mutants. Three single colonies of each *E. coli* strain were sequenced separately using Illumina Novaseq 6000: WT, Anc ΔL and ΔA. The outermost circle of the concentric circles is the reference sequence position coordinates of WT *E. coli MG1655* (GenBank number: GCF_000005845.2), and the second concentric circle is the gene distribution of the reference genome. Sequencing coverage for each isolate is plotted, according to color. Each SNP, represented by a vertical line colored according to isolate, is marked at the appropriate genomic position on the genomes in which it was found. Mutations that were common across all of the colonies sequenced for each *E. coli* strain were shown. (B) the two auxotrophic strains were grown in M9 medium, which was supplemented with 150 μM of the required focal amino acid (+), devoid of any amino acid supplementation (0), or supplemented with 150 μM of each of the 19 other proteinogenic amino acids (++). Growth of six replicates was determined turbidometrically (OD_600_) after 24 h of cultivation. Boxplots: Median (horizontal lines in boxes), interquartile range (boxes), 1.5-fold interquartile range (whiskers).

We genetically engineered *E. coli* MG1655 to generate two distinct auxotrophic genotypes with different metabolic capabilities. Specifically, the lysine auxotroph (ΔL) synthesizes arginine but requires lysine for growth, while the arginine auxotroph (ΔA) synthesizes lysine but requires arginine supplementation. Resequencing of the WT and auxotrophic strains confirmed the presence of several shared single nucleotide polymorphisms (SNPs) in auxotrophs inherited from the WT (Fig. 1A). To verify the specificity of the auxotrophic phenotypes, we hypothesized that each strain would be unable to grow in minimal medium lacking its required amino acid, and unable to synthesize the focal amino acid from other amino acid sources. Growth assays under three different conditions confirmed this hypothesis: each auxotroph required the focal amino acid for growth and could not substitute other amino acids (Fig. 1B).

**Fig. 2:**
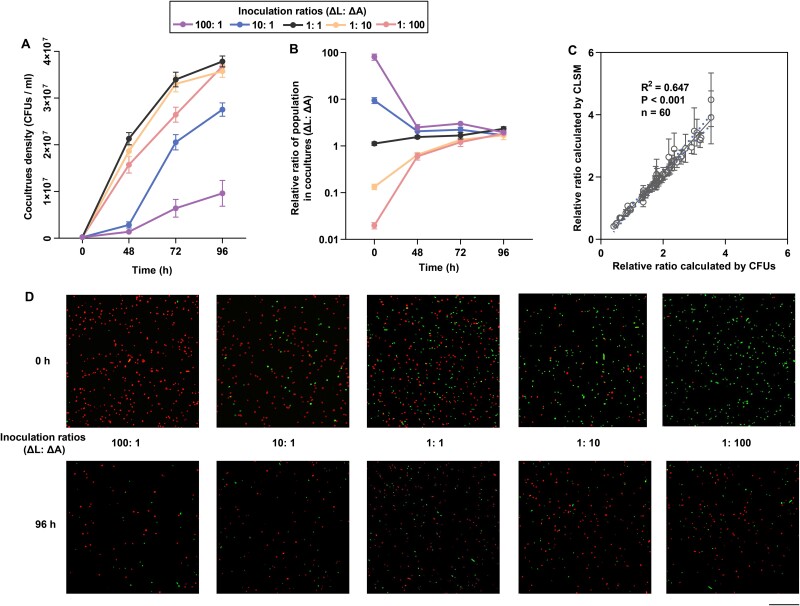
**Characterizing population dynamics of syntrophic *E. coli* cocultures over time.** Cocultures were inoculated at five different inoculation ratios (ΔL: ΔA). (A) the consortia density of cocultures was then measured by CFUs / ml over time. Mean ± s.e.m values (n = 4) were shown. (B) Strain relative ratios (ΔL: ΔA) in cocultures were estimated by measuring CFUs of ΔL relative to CFUs of ΔA. Mean ± s.e.m values (n = 4) were shown. (C) a linear regression was fitted to the relative ratios calculated by CFUs and CLSM method (area between dotted lines: ±95% confidence interval). The results of a Pearson correlation were shown. (D) CLSM micrographs of green- and red-fluorescing syntrophic cocultures over time.

When the two auxotrophs were washed free of supplements and mixed to form a coculture, both strains initially underwent residual growth using stored metabolites [31] and achieved coexistence. To investigate the impact of initial inoculation ratios on coculture dynamics, we set five initial ratios of ΔL: ΔA (100:1, 10:1, 1:10, 1:100, and 1:1) and monitored the population dynamics using CFUs/ml and CLSM (Fig. 2). Initial inoculation ratios significantly influenced maximal growth (Fig. 2A). Notably, consortia initiated with a higher proportion of ΔL relative to ΔA exhibited slower population growth (one-way ANOVA, *P* < .001, F = 81.65, df = 19, Fig. 2A). Despite differences in initial inoculation ratios, the relative frequencies of ΔL and ΔA converged to ~2:1 within 96 hours (Fig. 2B), indicating that each auxotroph exhibited higher relative fitness when initially rare, increasing in frequency until an equilibrium was reached. This phenomenon, consistent with “frequency-dependent selection” [32], suggests a mechanism for maintaining syntrophic stability in microbial communities [32]. A comparison of relative ratios calculated from CFU-based density estimates with those obtained from CLSM analysis showed strong consistency (Pearson correlation, R^2^ = 0.647, *P* < .001, n = 60, Fig. 2C), further validating the reliability of the CLSM method used in this study (Fig. 2D).

**2. Syntrophic cocultures are unstable in experimental evolution** 

**Fig. 3:**
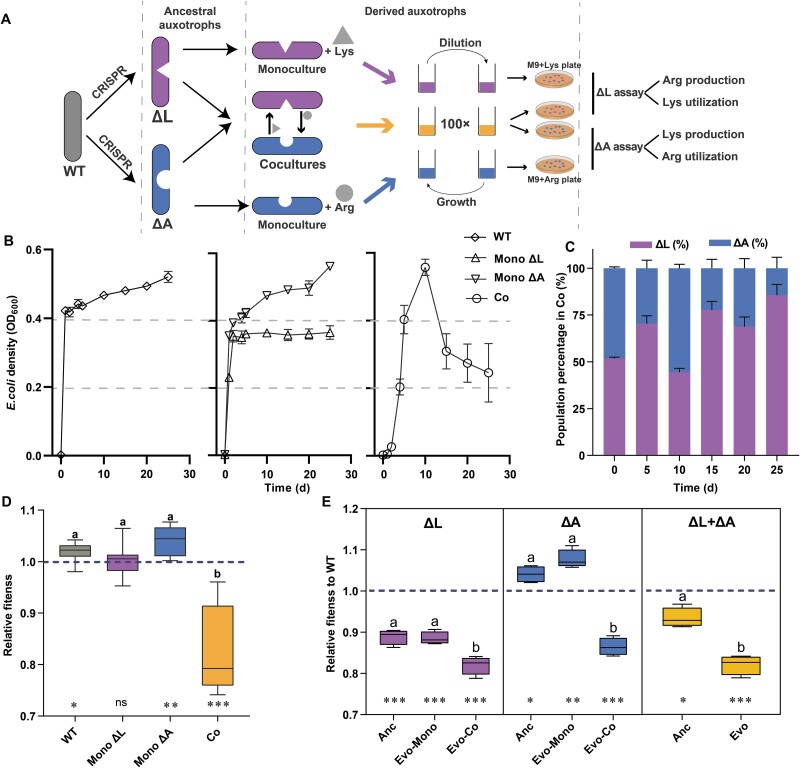
**Comparison of growth yield in the experimental evolution**. (A) Design of the experimental evolution. Modification of prototrophic *E. coli* (WT) by CRISPR-Cas9 genetic editing to create lysine auxotrophic and arginine auxotrophic *E. coli* (ΔL and ΔA). Three experimental treatments, i.e. monoculture of prototrophic strains (WT) without amino acids supplements, monocultures of auxotrophic strains (mono) supplemented with the lysine (+Lys) or arginine (+Arg), and cocultures of auxotrophs of *E. coli* (Co) without amino acids supplements, were serially propagated for 25 days in shaken liquid medium. Cocultures were transferred to fresh medium at a volume of 1:100 every 5 days. Derived auxotrophs from each transfer were isolated by selective plates and frozen for downstream assays. (B) Mean group density (mean ± s.e.m, n = 8) was quantified as optical density at 600 nm (OD_600_) over the course of the evolution experiment. (C)the population percentage of the two types calculated by CFUs/ml changes over time in cocultures (one-way repeated measures ANOVA: *P* < .0001, F (2.58, 18.07) = 19.15, partial η^2^ = 0.732). Data showed mean and s.e.m (n = 8). (D) Change in Darwinian fitness of derived populations relative to their evolutionary ancestors. Relative fitness is the net growth of derived consortia divided by the growth achieved by the corresponding ancestors during a certain period. Ancestral and derived populations were cultivated separated from each other. Different letters indicate significant differences (ANOVA followed by Bonferroni post hoc test: *P* < .001, F = 34.5, df = 28). The dashed line indicates equality in fitness between derived populations and the corresponding ancestors. Relative fitness of WT and monoculture of ΔA is significantly higher than 1 (one-sample t test, WT: *P* = .025, t = 2.842, n = 8, ΔA: *P* = .005, t = 4.1, n = 8), monoculture of ΔL has no significantly difference than 1 (one sample t test: *P* = .78, t = 0.265, n = 8), whereas the relative fitness of Co is lower different than 1(one sample t test: *P* < .001, t = 5.926, n = 8). (E) Competitive fitness of the amino acid auxotrophic strain or co-cultured consortia (ΔL + ΔA) relative to WT**.** Anc and Evo auxotrophs were competed against WT for 24 h in a minimal medium supplemented with required amino acids (150 μM), respectively. Different letters indicate significant differences (ANOVA followed by Bonferroni post hoc test, ΔL: *P* = .001, F = 16.5, df = 11; ΔA: *P* < .001, F = 110.1, df = 11. Independent sample t test, ΔL + ΔA, *P* < .001, t = 6.75, n = 4). The dashed line indicates equality in fitness between WT and the corresponding competitors. Asterisks indicate fitness values that were significantly different from 1 (i.e. WT fitness, one sample t-test, *P* < .05, n = 4).

Our laboratory evolution experiments were designed to investigate the potential mechanisms that destabilize bacterial syntrophic interactions. We established three treatment groups: (i) monocultures of prototrophic *E. coli* (WT), (ii) monocultures of the lysine and arginine auxotrophs (ΔL and ΔA, respectively), and (iii) cocultures of the auxotrophic strains at an initial 1:1 ratio, which was determined to optimize consortia growth (Fig. 3A). Cocultures were incubated in well-mixed minimal medium over 25 days with periodic dilutions every 5th day.

To assess growth dynamics across the groups, we measured the optical density (OD_600_). A marginal but significant increase in the WT populations was observed from the first growth cycle (Day 5) to the end of the experiment (paired sample t test: *P* = .001, t = 4.162, n = 8, Fig. 3B), and ΔA monocultures exhibited a 1.33-fold increase (*P* < .001, *t* = 8.26, n = 8, Fig. 3B). In contrast, ΔL monocultures showed no significant growth change (paired sample t test: *P* = .88, *t* = 0.16, n = 8, Fig. 3B). Similarly, cocultures exhibited no overall significant change in growth (Wilcoxon signed ranks test: *P* = .07, Z = −1.82, n = 8, Fig. 3B), but individual replicates showed high variability, with two coculture maintaining OD_600_ ~ 0.6 while the remaining six ranged from 0.07 to 0.23. Tracking the population density (CFUs/ml) of each strain over time revealed that ΔL consistently maintained higher density than ΔA in cocultures (Fig. S5).

Fitness comparisons revealed distinct evolutionary trends. WT and ΔA monocultures showed significant fitness increases relative to their ancestral populations (one-sample t test, WT: *P* = .025, t = 2.842, n = 8, ΔA: *P* = .005, t = 4.1, n = 8, Fig. 3D), whereas ΔL monocultures exhibited no significant fitness changes (*P* = .78, t = 0.265, n = 8, Fig. 3D). In cocultures, however, fitness decreased significantly (one-sample t test: *P* < .001, t = 5.926, n = 8, Fig. 3D). In competition experiments, both ΔL and ΔA, as well as their coculture consortia, exhibited significantly reduced fitness relative to WT after coculture evolution (ANOVA, ΔL: *P* = .001, F = 16.5, df = 11; ΔA: *P* < .001, F = 110.1, df = 11; independent t-test, ΔL + ΔA, *P* < .001, t = 6.75, n = 4, Fig. 3E). ΔL strains generated by *lysA* knockout showed significantly reduced growth compared to the WT (one-sample t-test: *P* < .001, t = −11.907, n = 4; Fig. 3E), whereas *argH* knockout significantly enhanced the growth of ΔA strains (*P* = .029, t = 3.96, n = 4; Fig. 3E).

Coculture stability was evaluated by monitoring whether strain ratios and total cell densities returned to pre-dilution levels after serial dilutions and reinoculation [9]. Stable cocultures are expected to restore initial ratios and reach or exceed pre-dilution cell densities upon reinoculation. In our experiments, cocultures deviated progressively from pre-dilution ratios, with ΔL increasingly dominating (one-way repeated measures ANOVA: *P* < .0001, F (2.58, 18.07) = 19.15, partial η^2^ = 0.732, Fig. 3C), and total cell densities declining (Fig. 3B), indicating instability. The relative ratio diverged from the optimal 2:1 ratio, likely due to adaptation and co-evolution.

**Fig. 4:**
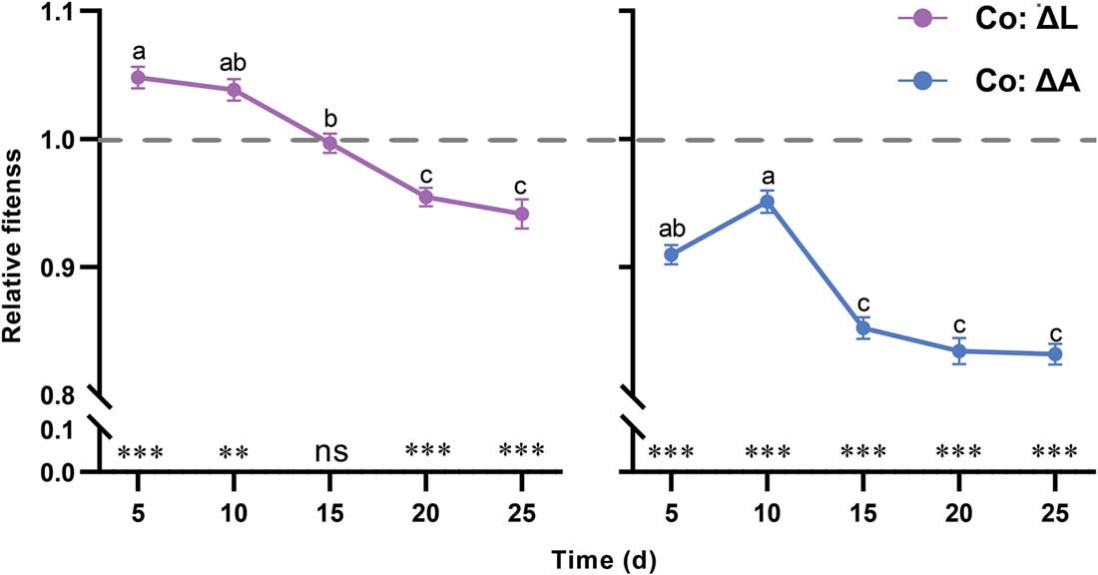
**Changes in the fitness of derived auxotrophs in cocultures relative to their evolutionary ancestors.** Relative fitness is the net growth of the derived population divided by the growth achieved by the corresponding ancestors (0 d) during a 24-h period. Ancestral and derived populations were cultivated separated from each other. Different letters indicate significant differences (one-way repeated measures ANOVA for ΔL strains: *P* < .0001, F (4, 28) = 26.851, partial η^2^ = 0.793; for ΔA strains: *P* < .0001, F (4, 28) = 33.223, partial η^2^ = 0.826). The dashed line indicates equality in fitness between derived auxotrophs and their corresponding ancestors. Mean ± s.e.m values (n = 8) were shown. Asterisks indicate fitness values that were significantly different from 1 (one-sample t test for derived ΔL: 5th day: *P* = .001, t = 5.562, 10th day: *P* = .003, t = 4.460, 15th day: *P* = .663, t = −0.455, 20th day: *P* < .001, t = −6.206, 25th day: *P* = .001, t = −5.114; for derived ΔA: *P* ≤ .001, t_5d_ = −12.070, t_10d_ = −5.545, t_15d_ = −17.527, t_20d_ = −16.239, t_25d_ = −20.648).

To quantify evolutionary changes in cocultures, fitness measurements were taken at various intervals by comparing biomass accumulation of derived strains to their ancestral counterparts. Derived ΔL populations initially exceeded ancestral ΔL fitness on Days 5 and 10 but showed fitness declines by Day 20. In contrast, derived ΔA populations consistently exhibited lower fitness compared to their ancestral ΔA populations throughout the experiment (Fig. 4).

**3. Auxotrophs adjust their metabolite utilization according to the metabolite production of their partner** 

**Fig. 5:**
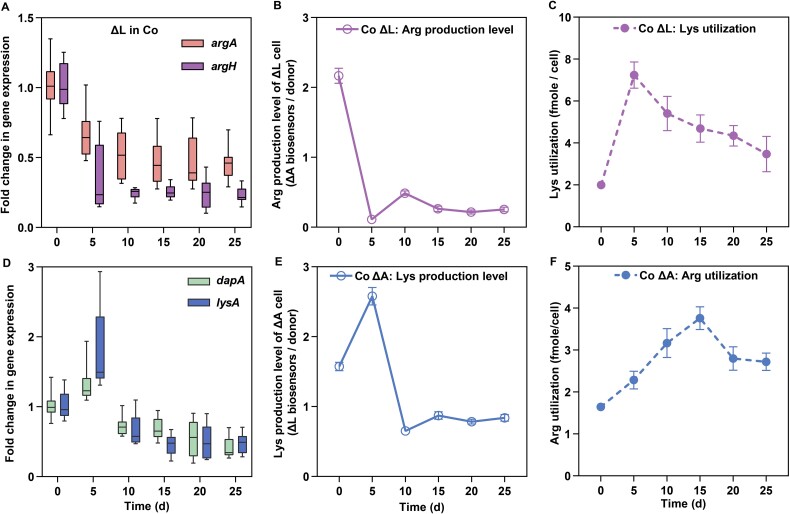
**Temporal dynamics of gene expression, metabolite production, and metabolite utilization in auxotrophic strains during coculture.** (A) Fold changes in gene expression of *argA* and *argH*, key genes involved in arginine biosynthesis in ΔL strains, across various time points in coculture, relative to their ancestral ΔL strains. (B) Arginine production levels in individual ΔL cells over the course of coculture, as measured by the biosensor method. Mean ± s.e.m (n = 8) values were shown. (C) Lysine utilization per ΔL cell during coculture. Mean ± s.e.m (n = 8) values were shown. (D) Fold changes in gene expression of *dapA* and *lysA*, key genes involved in lysine biosynthesis in ΔA strains, across various time points in coculture, relative to their ancestral ΔA strains. (E) Lysine production levels in individual ΔA cells during co-culture, as measured by the biosensor method. Mean ± s.e.m (n = 8) values were shown. (F) Arginine utilization rates by individual ΔA cells during co-culture. Mean ± s.e.m (n = 8) values were shown. All data at 0d represented the ancestral level of auxotrophs.

The instability of syntrophic cocultures can be attributed to multiple factors, including the diffusion of shared metabolites and the metabolic demand of each auxotrophic strain [9]. We assessed metabolite diffusion by measuring production levels using biosensor assays (Fig. 5B, E) and analyzing the expression of genes associated with metabolite synthesis (Fig. 5A, D). Derived ΔL cells from cocultures showed a significant reduction in arginine production compared to their ancestral counterparts (One-way Repeated Measures ANOVA: *P* < .000067, F (1.262, 17.933) = 61.195, partial η^2^ = 0.911; Fig. 5B). This reduction aligned with decreased expression of *argA* and *argH* in derived ΔL cells (*argA*: *P* = .036, F (1.673, 5.02) = 7.157, partial η^2^ = 0.705; *argH*: *P* = .003, F (1.181, 3.544) = 52.913, partial η^2^ = 0.946; Fig. 5A). Pearson correlation analysis revealed a strong positive relationship between *argA*/*argH* expression trends and arginine production levels measured via biosensor assays (Table 1). In contrast, derived ΔA cells from cocultures initially exhibited a 64% increase in lysine production compared to ancestral ΔA cells by Day 5 (One-way Repeated Measures ANOVA: *P* < .000067, F (1.780, 12.360) = 27.299, partial η^2^ = 0.796; Fig. 5E). However, lysine production declined significantly in later stages, reaching levels 50% lower than the ancestral ΔA cells (Fig. 5E). Expression analysis showed minimal changes in *dapA* (*P* = .047, F (1.359, 4.078) = 7.54, partial η^2^ = 0.715; Fig. 5D) but a significant elevation in *lysA* expression on Day 5 (paired sample t-test: *P* = .002, t = 4.076, n = 12; Fig. 5D), correlating with increased lysine production (Table 1). In monocultures, the production of both amino acids remained stable throughout the experiment (arginine production of ΔL: *P* = .835, F (4.780, 14.339) = 0.399, partial η^2^ = 0.117; lysine production of ΔA: *P* = .783, F (5, 15) = 0.484, partial η^2^ = 0.139, Fig. S6A).

**Table 1:** Coefficient of determination (R^2^) versus *P*-value of a calibration curve generated from the relative change in expression of key genes for amino acid synthesis versus the relative change in individual amino acid production levels detected by the biosensor method.

Variable	R^2^	*P*-value
*argA* expression & arginine production level	0.8120	0.0142
*argH* expression & arginine production level	0.9387	0.0014
*dapA* expression & lysine production level	0.6340	0.0580
*lysA* expression & lysine production level	0.9426	0.0013

**Fig. 6:**
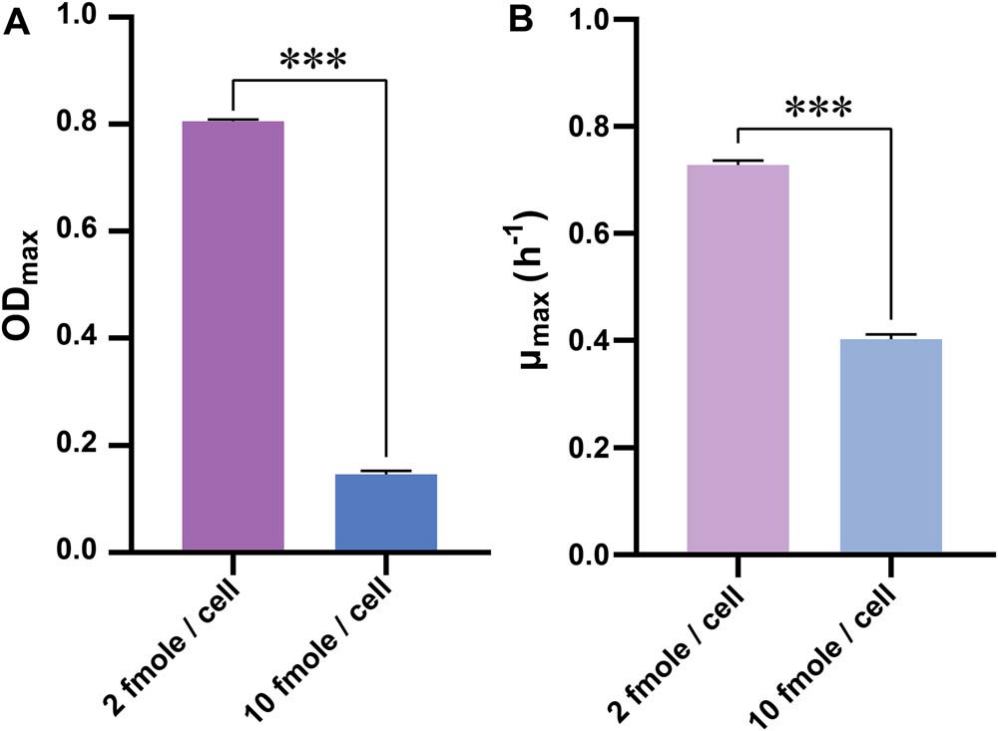
**Comparison in maximum population density (A) or growth rate (B) of ΔL strain with different lysine utilization.** The ΔL cells with higher lysine utilization get lower population density and growth rate in the M9 medium with the same concentration (50 μM) of input lysine. Mean ± s.e.m values (n = 4) were shown. Asterisks indicated significant differences among groups (independent t tests: OD_max_: ***P < .001, t = 79.37, μ_max_: ***P < .001, t = 26.15).

Amino acid utilization by ΔL and ΔA cells in cocultures showed significant changes over time, reflecting their adaptation to dynamic resource availability. Lysine utilization by ΔL cells increased significantly (Friedman rank test: *P* < .000067, χ^2^ = 31, df = 5; Fig. 5C), while arginine utilization by ΔA cells also showed significant changes (One-way Repeated Measures ANOVA: *P* < .000067, F (5, 35) = 8.371, partial η^2^ = 0.545; Fig. 5F). In monoculture, however, lysine and arginine utilization remained stable (Friedman rank test: lysine utilization of ΔL: *P* = .407, χ^2^ = 5.071, df = 5; arginine utilization for ΔA: *P* = .859, χ^2^ = 1.929, df = 5, Fig. S6B). Amino acid utilization reflects the metabolic demand of auxotrophic cells for limited resources [19], which in turn, influences population density. Increased lysine utilization by ΔL cells correlated with reduced ΔL population density and growth rates under lysine-limited conditions (Fig. 6).

**Fig. 7:**
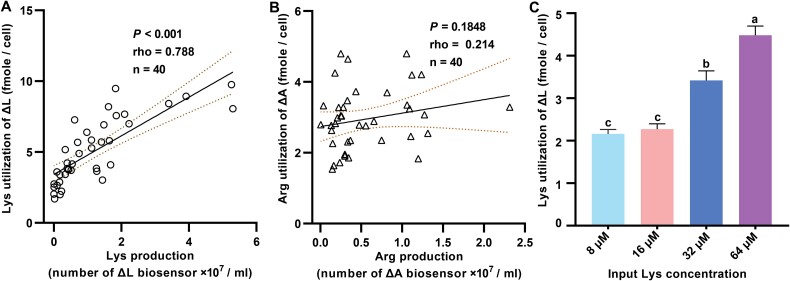
**The variation in metabolite utilization as an adaption to partner production.** (A) The lysine utilization of lysine auxotrophic cells was significantly correlated with the lysine production from partner populations in cocultures. A linear regression was fitted to the data (black line; area between dotted lines: ±95% confidence interval). The results of a Pearson correlation were shown. (B) The arginine production from the ΔL population (x-axis) was not related to the arginine utilization of the ΔA cell (y-axis). A linear regression was fitted to the data (black line; area between dotted lines: ±95% confidence interval). Results of Pearson correlations were shown. (C) The effect of different concentrations of exogenous amino acids on lysine utilization. Detailed experimental procedures were provided in supplementary methods. Mean ± s.e.m values were shown. Different letters indicated significant differences among groups (one-way ANOVA: *P* < .001, F = 35.326, df = 31).

Lysine production by the ΔA population positively correlated with lysine utilization by ΔL cells in cocultures (Pearson’s correlation: *P* < .001, r = 0.79, n = 40; Fig. 7A). However, no significant correlation was observed between arginine utilization by ΔA cells and arginine production by ΔL populations (Fig. 7B). The “lysine utilization variability assay” (Supplementary method) further confirmed that higher environmental lysine concentrations led to increased lysine utilization by ΔL cells (Fig. 7C). These findings suggest that auxotrophs adjust their metabolite utilization in response to the metabolite production of their syntrophic partners, highlighting the dynamic interplay between resource production and demand in shaping coculture stability.

## Discussion

Metabolite exchange is crucial for sustaining microbial consortia in resource-limited environments [33]. The exchange of metabolic by-products by cells can drive the evolution of bacterial lineages that engage in costly, mutually beneficial cooperation. Such cooperation often arises within a spatially structured environment, where cells aggregate into clusters, thus promoting the development of complex interdependencies among microbes with distinct genotypes [10, 11, 14, 34]. However, in non-spatially structured environments, there are a lack of clear benefits to individuals to enhance the fitness of others with non-cooperating genotypes under positive selection [14]. This study examines how auxotrophic *E. coli* strains establish and destabilize syntrophic interactions in a non-spatially structured environment, providing insights into the ecological and evolutionary dynamics driving these interactions.

The invasion-from-rare experiment showed that auxotrophic strains rapidly form syntrophic consortia through metabolite exchange, with frequency-dependent selection stabilizing the strain ratios at a 2:1 equilibrium within 96 hours (Fig. 2B). This outcome aligns with predictions of stable selection in cooperative systems [32]. However, laboratory evolution experiments revealed the evolutionary instability of these interactions. After 25 days, most cocultures ceased growth, with ΔL populations reaching a peak frequency of 98%. Furthermore, the overall fitness of the consortia and the individual fitness of both auxotrophs declined over time (Fig. 3D, 3E, and Fig. 4), indicating that syntrophy between ΔL and ΔA strains is not evolutionary stable and that a “selfish” phenotype can invade and disrupt the cooperative population. The production levels of exchanged metabolites reflect the degree of cooperation between auxotrophs [10]. Using the metabolic production of ancestral strains as a baseline, we found that derived ΔL cells adopted a selfish strategy by significantly reducing arginine production, corresponding to the downregulation of *argA* and *argH* expression (Fig. 5A, B). Conversely, ΔA cells initially displayed cooperative traits by increasing lysine production during the first transfer cycle, supported by the upregulation of *lysA* and *dapA* (Fig. 5D, E). However, the selfish behavior of ΔL cells was a key factor in this instability, exploiting the lysine produced by ΔA strains while reducing their own contribution to arginine exchange. Such selfish phenotypes exhibit higher relative fitness when rare, especially in microbial communities [35]. Thus, negative frequency-dependent selection can predict the rapid invasion of non-cooperative individuals into cooperative populations [35]. When “selfish” behavior persists in a largely cooperative population, selection will favor mechanisms in cooperators (so-called resistance to non-cooperators) that reduce the degree to which they can be exploited [35]. For example, some angiosperm plants reduce their exploitation by less-cooperative individual pollinator insects by limiting the nectar rewards for pollination [36]. Over time, derived ΔA cells attempted to resist exploitation by reducing lysine production (Fig. 5D, E), but this resistance failed to stabilize the syntrophic interaction.

The fitness of each auxotroph was strongly influenced by their metabolite utilization patterns (Fig. 6). Previous studies have used metabolite utilization factors in determining the evolutionary fates of microbial cooperation [19]. Here, we highlight the effects of metabolite utilization in the evolution of cross-feeding between different *E. coli* strains. Both strains adapted their amino acid utilization in response to the local metabolite environment created by their partner (Fig. 5C, F), a phenomenon absent in monocultures. We conclude that coexisting auxotrophs adapt to the local metabolite environment created by their partners through variations in amino acid utilization. However, our two auxotrophic strains varied in their response to environmental changes. ΔL cells displayed a dynamic relationship between lysine utilization and lysine production by ΔA strains (Fig. 7A), enabling ΔL populations to maintain relative stability (Fig. S5) even under declining lysine availability (Fig. S7). This metabolic plasticity allowed ΔL cells to mediate the costs of resistance strategies employed by ΔA cells, thereby outcompeting their cooperative partner. In contrast, no correlation was observed between arginine utilization by ΔA cells and arginine production by ΔL populations (Fig. 7B), reflecting the inability of ΔA cells to adaptively regulate resource utilization. This lack of plasticity subjected ΔA strains to negative selection when selfish variants emerged, further destabilizing the interaction. The observed asymmetry in metabolic responses underscores the critical role of plasticity in shaping fitness outcomes. ΔL cells, through adaptive utilization of lysine, consistently exploited ΔA cells, transforming a cooperative interaction into one dominated by selfish competition. Similar asymmetric coevolutionary patterns have been documented in natural microbial communities, where metabolic specialization often favors the more adaptive partner [8, 19].

The evolution of cooperative bacterial populations raises fundamental questions for evolutionary biology: how do auxotrophic microorganisms establish stable nutritional codependence? Our results highlight two key factors destabilizing syntrophic relationships: the emergence of selfish phenotypes (e.g. derived ΔL cells) and asymmetric responses to environmental change. The selfish behavior of ΔL cells undermined syntrophic cooperation, while ΔA cells’ resistance strategies (e.g. reduced lysine production) proved ineffective due to ΔL cells’ rapid adaptive responses. Consequently, low levels of metabolite exchange by both strains were insufficient to sustain stable interactions. These findings emphasize the delicate balance between cooperation and competition in microbial systems. Resistance to selfish behaviors can stabilize cooperation in some cases [35], but specific metabolic patterns, as observed here, may override such strategies, leading to the collapse of mutually beneficial interactions. These findings provide a framework for exploring the evolution of microbial cooperation and have practical implications for designing stable synthetic consortia for biotechnological applications. In the future, we will integrate genomic data, such as whole-genome sequencing of ancestral and evolved strains, to gain deeper insights into the underlying evolutionary mechanisms.

## Supplementary Material

Supplementary_Information_ycaf011

## Data Availability

Data used in this manuscript are available on Figshare: https://doi.org/10.6084/m9.figshare.26839096.v2. Additional genomic data generated in this study have been deposited under the BioProject PRJNA1203638 in the NCBI database.
