# Supplementary material for: Asymmetric metabolic adaptations undermine stability in microbial syntrophy
Source: ISME Commun. 2025 Jan 25;5(1):ycaf011. doi: 10.1093/ismeco/ycaf011 (PMC11815887; doi:10.1093/ismeco/ycaf011)
Supplement: Supplementary_Information_ycaf011 [file supplementary_information_ycaf011.pdf]

Supplementary Information for

Asymmetric Metabolic Adaptations Undermine Stability in Microbial Syntrophy

Nan Ye[1,2†], Zhi-Chun Yang[1,2†], Zhuang-Dong Bai[1,2*]

1. School of Ecology and Environment, Northwestern Polytechnical University, Xi'an 710129, China

2. Shaanxi Key Laboratory of Qinling Ecological Intelligent Monitoring and Protection, School of Ecology and Environment, Northwestern Polytechnical University, Xi'an 710129, China

†Authors contributed equally

*For Correspondence: baizhuangdong@163.com

This PDF file includes:

Figures S1 to S8
Supplementation methods
Supplementation tables 1 to 3

Supplementary figures

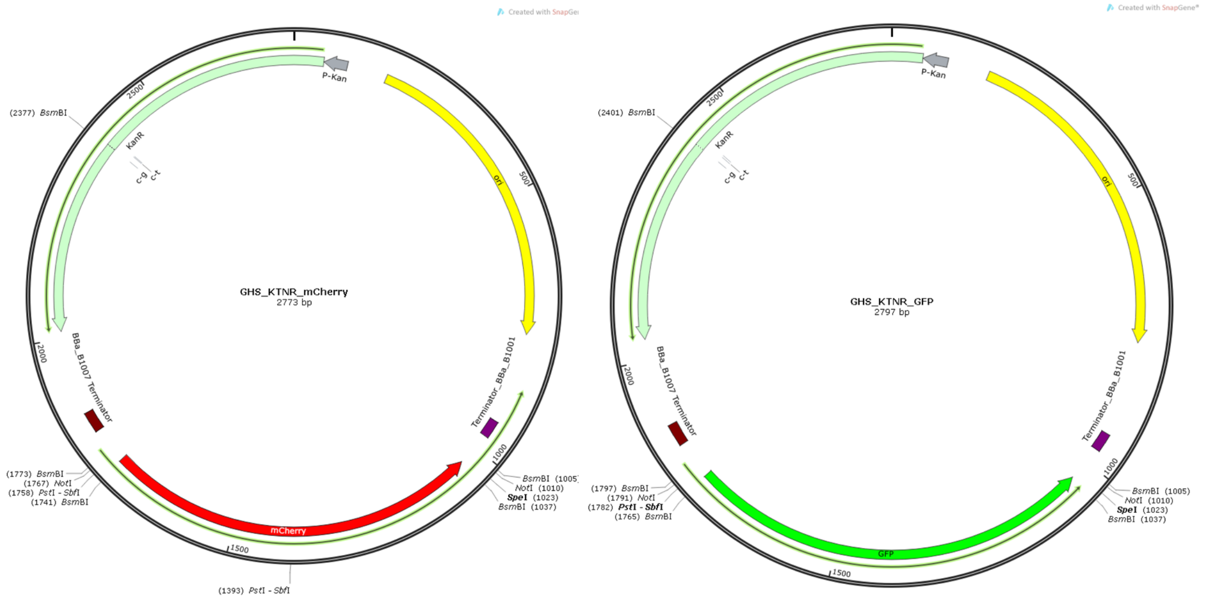

Fig. S1| Fluorescent plasmid profile.

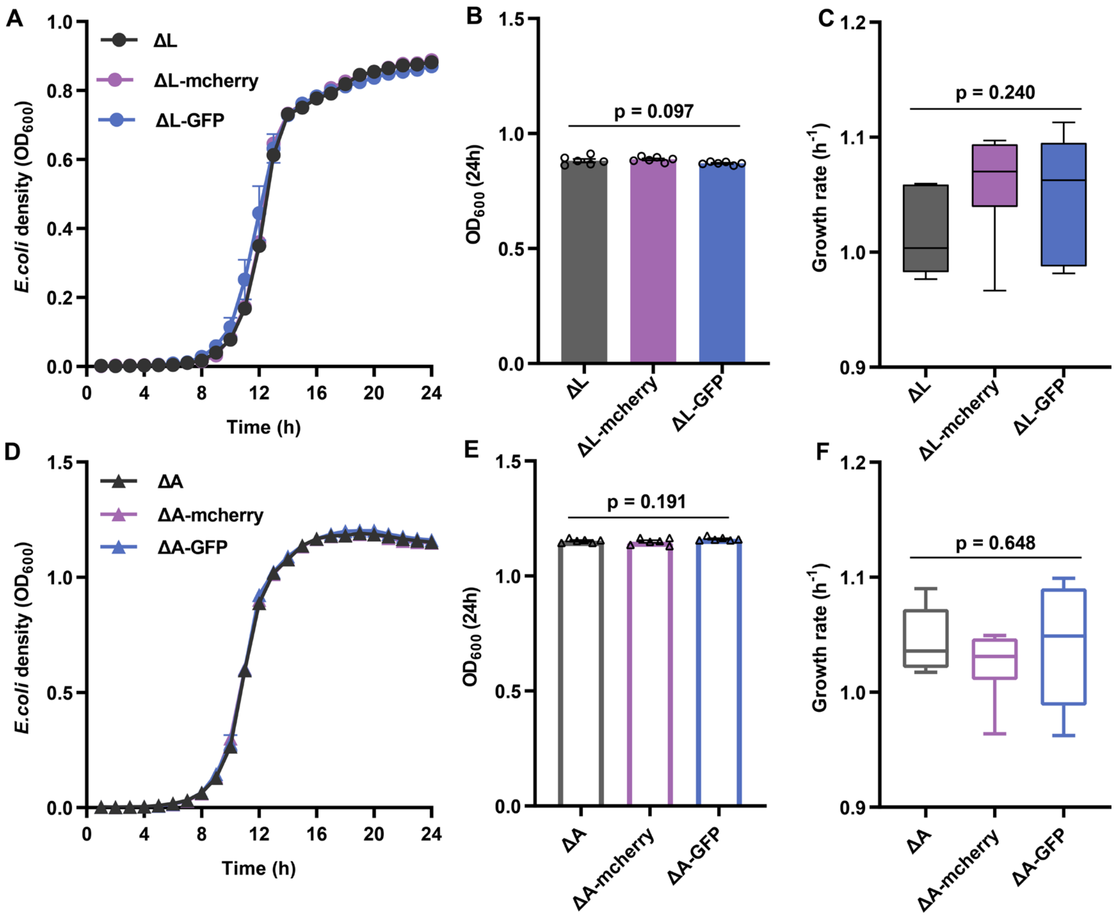

Fig. S2| Assessment of the expression of two fluorescence proteins on auxotrophic cell growth. (A, D). Comparison of cell growth curves, (B, E). OD_{600} values at 24 h and (C, F). growth rate (h^{-1}) of these two strains carrying different fluorescent proteins. Mean ± s.e.m values (n = 6) were shown. One-way ANOVA, followed by Bonferroni's multiple comparisons test with 95% confidence intervals were shown.

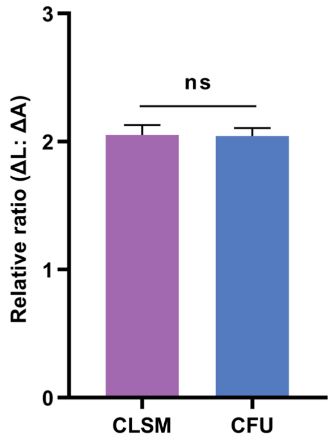

Fig. S3| Comparison of the relative ratio of CLSM acquisitions to CFU acquisitions. ΔL and ΔA were co-cultured in M9 medium at an initial ratio of 1:1 for 72 h. The relative ratios of ΔL: ΔA at 72 h were calculated according to the CLSM method (**Materials and Methods**, 'Confocal laser scanning microscopy', purple bar) and CFU method (**Materials and Methods**, 'strains and culture conditions', blue bar), respectively. There was no significant difference between the results obtained by the CLSM and CFU methods (Independent sample t-test, $P = 0.9282$, $t = 0.0918$, $n = 8$).

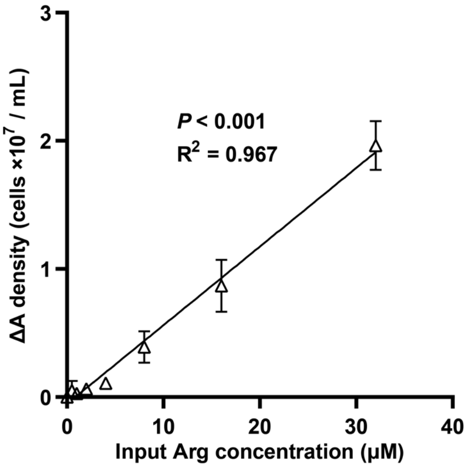

Fig. S4| Typical calculation of arginine utilization per individual of arginine auxotrophic strains. Calculation of arginine utilization of ΔA cell by fitting the saturated bacterial density of axuotrophs (y axis) at different supplemented arginine concentrations (x axis). The result of fitting coefficient was shown (area between dashed lines: ±95% confidence interval, n = 8). And utilization per cell in a saturated culture was quantified from 1/slope.

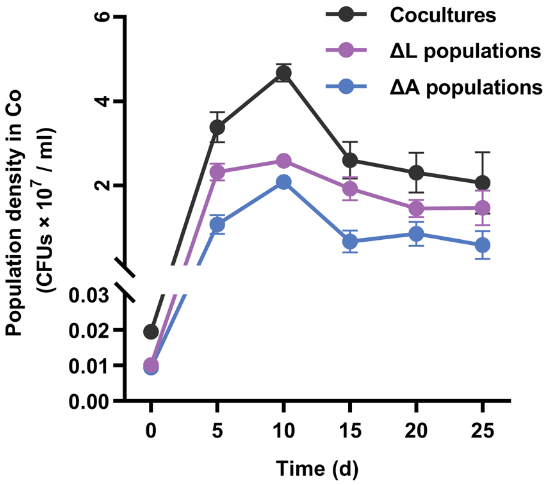

Fig. S5| Mean group density (mean ± s.e.m, n = 8) quantified as CFUs/ml over the course of the laboratory evolution experiment. To compare the population densities of the two *E. coli* strains (ΔL and ΔA), we applied a linear mixed-effects model (LMM) using the *lme4* package in R. The model was fitted using Restricted Maximum Likelihood (REML), and significance of the fixed effect (strain type) was assessed using Wald Z-tests. Model assumptions, including normality of residuals and random effects, were checked during analysis. The population density of ΔL was significantly higher than that of ΔA in cocultures (Linear mixed-effects model, $\beta = 0.746 \pm 0.185$, $z = 4.027$, $P < 0.001$).

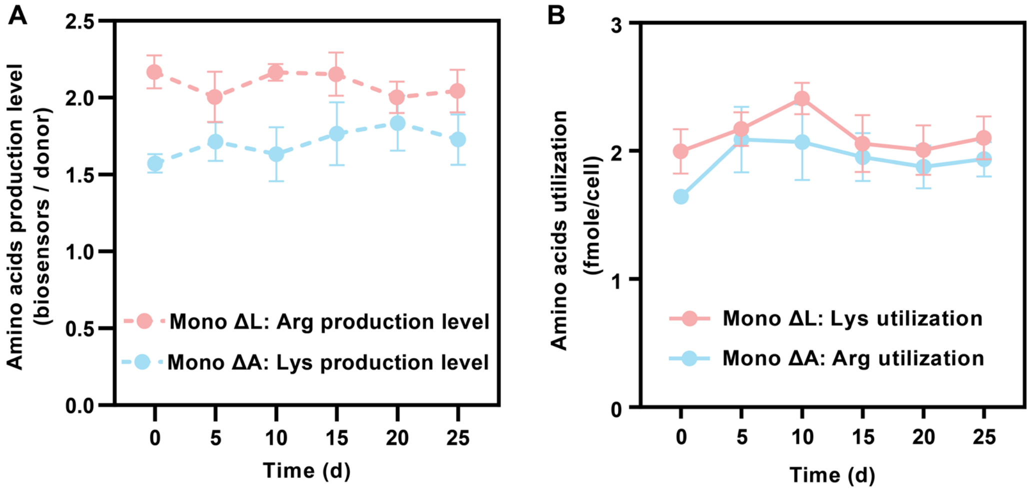

Fig. S6| Metabolic production and consumption dynamics for two auxotrophic individuals in monoculture. (A) Dynamics of arginine (pink) and lysine production level (blue) by ΔL cell and ΔA cell in monoculture. The data at 0d represented the ancestral amino acid production levels. Mean ± s.e.m values (n = 4) were shown. (B) Dynamics of lysine (pink) and arginine utilization (blue) by ΔL cell and ΔA cell in monoculture. The data at 0d represented the ancestral amino acid utilizing level. Mean ± s.e.m (n = 8) values were shown.

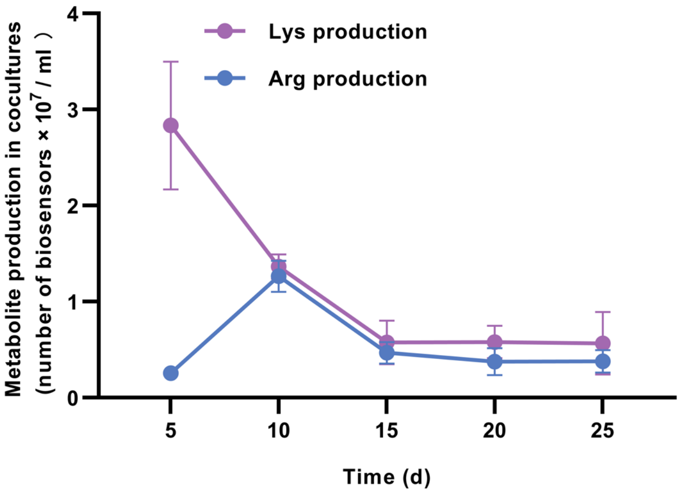

Fig. S7| The metabolite production in population level in cocultures at different moments. We multiplied the amino acid production level per cell with the number of auxotrophic cells to obtain the focused lysine (purple circle) or arginine (blue circle) production available in the cross-feeding system. Mean ± s.e.m (standard error) values (n = 8) were shown.

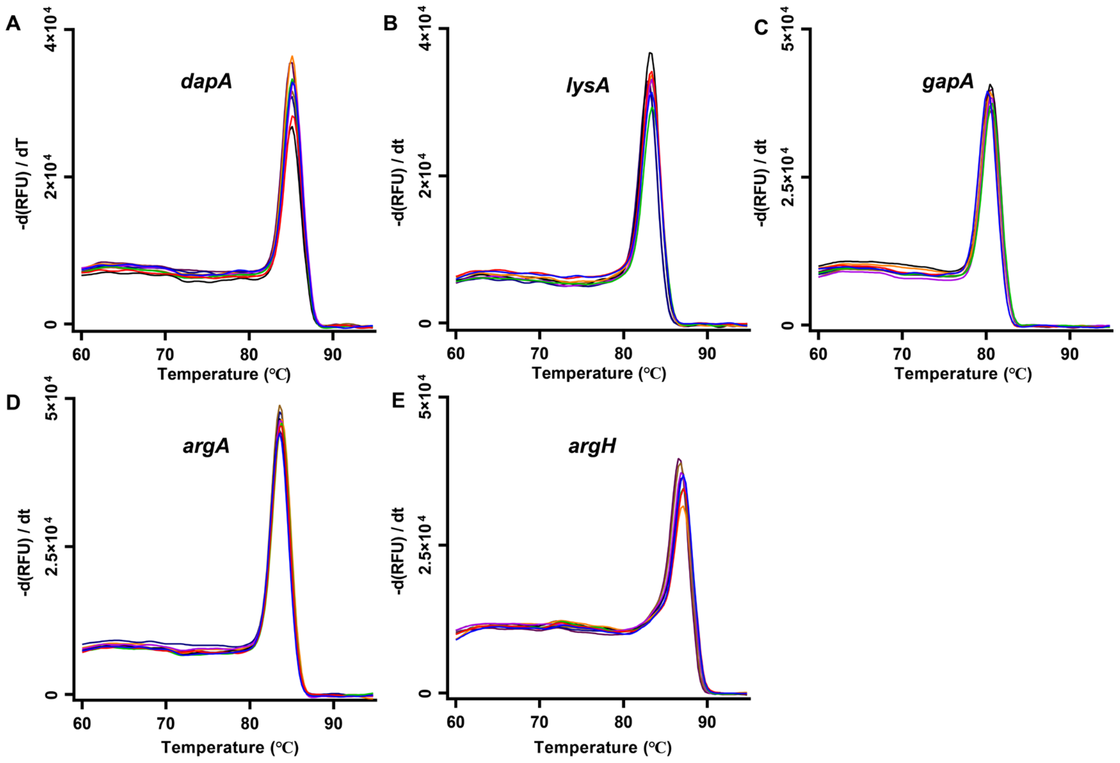

Fig. S8| Melting curves for the genes (A: *dapA*, B: *lysA*, B: *gapA*, D: *argA*, E: *argH*).

Supplementation methods.

Fluorescently labeled auxotrophic strains growth assay

Auxotrophic strains, labelled with or without fluorescent markers (mCherry and GFP), were pre-cultured in M9 medium supplemented with 150 μM lysine or arginine, harvested at the late-exponential phase, and washed three times in M9 medium by centrifugation at 12000 rpm for 5 minutes. Cell densities were determined by optical density readings (Biotek Synergy Neo2, Vermont, America) at 600 nm (OD_{600}). Precultures were diluted to OD_{600} ~ 0.1, and 2μl of the preculture dilutions were cultivated in 198 μl of amino acid-containing M9 medium (lysine or arginine, 150 μM). Kanamycin (50 μg/mL) was also added to the medium containing fluorescently labelled auxotrophic strains. The optical density at 600 nm (OD_{600}) of cultures were measured every 1 hour in microplate reader (Biotek Synergy Neo2, Vermont, America). This experiment was replicated six times and results were shown in Fig. S2.

Whole-genome sequencing

Resequencing was used to explore the single nucleotide polymorphisms (SNPs) and small insertions/deletions (indels) with high confidence in the ΔL and ΔA. Three ancestral ΔL or ΔA colonies used for sequencing were single colonies obtained after CRISPR-Cas 9 modification. Additionally, three initial wild type colonies were sequenced for reference. Genome DNA was extracted using Bacterial DNA Extraction Kit (FINDROP, Guangzhou, China). Sequencing libraries were generated using the ALFA-SEQ DNA Library Prep Kit (FINDROP, Guangzhou, China). The library quality was assessed on the Qubit 4.0 Fluorometer (Life Technologies, Grand Island, NY) and Qsep400 High-Throughput Nucleic Acid Protein Analysis system (Houze Biological Technology Co, Hangzhou, China). Sequencing was conducted on an Illumina Novaseq6000 platform (Guangdong Magigene Biotechnology Co., Ltd., Guangzhou, China) with 150 bp paired-end reads. After sequencing, the raw data underwent quality control, including removal of low-quality reads and adapter trimming, to generate high-

quality clean data. This step was performed using the standard quality filtering thresholds: Q20 and Q30. The clean data were then aligned to a reference genome (GenBank number: GCF_000005845.2) using BWA (v0.7.17) and SAMtools (v1.7) to obtain sequencing coverage and determine the close relationship between the sample and the reference sequence. Single nucleotide polymorphisms (SNPs) and insertions/deletions (InDels) were identified using SAMtools. These variants were annotated to understand their potential impacts using established tools. Structural variants (SVs) were detected using BreakDancer, and we employed Circos to visualize the distribution of SNPs and INDELs across the genome. Mapping quality was assessed to ensure good alignment between reads and the reference genome. Average coverage depth and coverage rates at >=1x, >=10x, >=50x, and >=100x were calculated to verify the depth of coverage. The mapping rate of reads was consistently above 99%, indicating high-quality sequence alignment.

RNA extraction and cDNA synthesis

Total RNA was extracted from ΔL and ΔA strains pre-cultured from single colonies isolated from cocultures at various coculture transfer points (Days 0, 5, 10, 15, 20, and 25). Pre-cultures were grown in M9 medium supplemented with 150 μM lysine or arginine at 37°C, 220 rpm for 12 hours. Cells were pelleted by centrifugation at 12,000 rpm for 2 minutes at 4 °C and lysed using RNAiso Plus reagent (Takara) according to the manufacturer's protocol. RNA extraction involved separation of the aqueous phase with chloroform, precipitation with isopropanol, and washing with 75% ethanol. The RNA pellet was resuspended in RNase-free water and treated with DNase I (RNase-free, Thermo Fisher Scientific) to eliminate residual genomic DNA. RNA concentration and purity were determined using a NanoDrop spectrophotometer (Thermo Fisher Scientific), and samples with A260/A280 ratios close to 2.0 were retained for further analysis. For cDNA synthesis, 500 ng of RNA was reverse-transcribed using the GoScript™ Reverse Transcription Mix Kit (Promega) according

to the manufacturer's instructions. The resulting cDNA was stored at -20°C for subsequent qPCR analysis.

Quantitative PCR (qPCR)

qPCR was used to measure the relative expression of key biosynthetic genes involved in lysine and arginine production in auxotrophic strains at each coculture transfer point. In ΔA, lysine biosynthesis was assessed by quantifying expression of *dapA* (encoding dihydrodipicolinate synthase, catalyzing the first step of lysine biosynthesis) and *lysA* (encoding meso-diaminopimelate decarboxylase, catalyzing the final step of lysine synthesis). In ΔL, arginine biosynthesis was evaluated by measuring *argA* (encoding the enzyme for the initial step in arginine biosynthesis) and *argH* (encoding the enzyme catalyzing the final step, converting argininosuccinate to arginine). The housekeeping gene *gapA* was used as an internal control for normalization. Primers were designed using Primer3 (http://primer3.ut.ee/) and had the following sequences:

dapA: forward 5'-GCGGTCATGGGGTTATTTCC-3'**,** reverse 5'-ACTGTCGGTGATTGGTGTCA-3'.

lysA: forward 5'-CGTTAATCCGGGGTTTGGTC-3', reverse 5'-TCAACGCCAGAACCAATGTG-3'

argA: forward 5'-CGGGAACATTGCAACTGGAT-3'**,** reverse 5'-CGGCCCCATTAGCACTATTG-3'.

argH: forward 5'-TTACCCAGGCAGCAGATCAA-3'**,** reverse 5'-GCGAACATCTTCCAGCAACA-3'.

gapA: forward 5'-GAAATGGGACGAAGTTGGTG-3'**,** reverse 5'-AACCACTTTCTTCGCACCAG-3'.

qPCR reactions were performed in 10 µL reaction volumes containing 5 µL SYBR Green Master Mix (QIAGEN), 0.7 µL of each primer (200 nM final concentration), 1 µL of cDNA template, and 2.6 µL of RNase-free water. Each sample was run in

triplicate using an Applied Biosystems QuantStudio 5 real-time PCR system (Thermo Fisher Scientific). The cycling conditions were as follows: initial denaturation at 95°C for 2 minutes, followed by 40 cycles of 95°C for 5 seconds and 60°C for 10 seconds. Melt curve analysis was performed to confirm product specificity (Fig. S8).

Threshold cycle (C_T) values were used to calculate the gene expression. Relative fold changes in gene expression changes were calculated using the $2^{-\Delta\Delta CT}$ method [1], with day 0 serving as the control reference point.

Lysine utilization variability assay.

To confirm that lysine utilization by ΔL cells varies with environmental lysine concentration, eight derived ΔL clones were selected for testing. M9 medium was supplemented with one of four lysine concentrations (C) (8 μM,16 μM,32 μM and 64μM). For each treatment, ΔL cells were inoculated at the same initial density (N_0) and incubated until reaching the saturation threshold (N_t). The average individual lysine utilization was calculated based on the ratio of lysine concentration to bacterial growth (i.e., $\frac{C}{Nt-N0}$). To ensure that the exogenous amino acids were exhausted, an equal volume of 2×M9 medium was added to the supernatant of the cultured bacteria, followed by the addition of fresh ΔL cells. After 24 hours of incubation, no increase in bacterial density was observed in any treatment, confirming that the exogenous lysine in the original medium had been fully exhausted.

Supplementation table S1

Recombinant DNA	Description	source
GSH_KTNR_GFP	express the green fluorescent protein and kanamycin resistance	This study
GSH_KTNR_mCherry	express the red fluorescent protein and kanamycin resistance	This study
Cas9	cas9 plasmid to knockout gene	[2]
SgRNA	SgRNA to knockout gene	[2]
Donor DNA	500 bp fragments at each end of *lysA* or *argH* ligated together	Amplification by PCR in *E. coli MG1655*

Supplementation table S2. Primer information of CRISPR-Cas9 method.

Primer name	Sequences (5'- 3')
up lysA-F	CCCCGCTTGTAAATGCCCAC
up lysA-R	CAACCAGCGACTAACCGCAGAACAAACTCCAGATAAGTGCTTTTTATG
down lysA-F	GCACTTATCTGGAGTTTGTTCTGCGGTTAGTCGCTGGTTGC
down lysA-R	TTCAGTCGCCACCGTTTCCC
up argH-F	GTTGGGCTGCCATTTTGCGATATC
up argH-R	ATTTATACATATAAATGTTCAACTCTGTTTCCTTATTTTGAAATTCAATGCCG
down argH-F	CAAAATAAGGAAACAGAGTTGAACATTTATATGTATAAATTTGAGCCTGG
down argH-R	ACAGCCAGCGCTGGCAGTAAAG
argH-N20-F	TTACCCAGGCAGCAGATCAAGTTTTAGAGCTAGAAATAG
argH-N20-R	TTGATCTGCTGCCTGGGTAAACTAGTATTATACCTAGG
lysA-N20-F	TCGGTGCTGAACAGTGAATGGTTTTAGAGCTAGAAATAG
lysA-N20-R	CATTCACTGTTCAGCACCGAACTAGTATTATACCTAGG

Note: Forward and reverse primers for a 500 bp DNA fragment upstream of *lysA:* 'up lysA-F' and 'up lysA-

R'. Forward and reverse primers for the 500 bp DNA fragment downstream of *lysA:* 'down lysA-F' and 'down lysA-R', and so on. Forward and reverse primers of the *lysA* that bind to SgRNA: 'lysA-N20-F' and 'lysA-N20-R', and so on.

Supplementation table S3

Reagents and chemicals	Source/supplier	Catalog number
M9 Minimal Salts	Sangon Biotech	A507024-0250
Glucose	Sangon Biotech	A610219-0500
$MgSO_4 \cdot 7H_2O$	Sangon Biotech	A610329-0500
$FeSO_4 \cdot 7H_2O$	Sangon Biotech	A600461-0500
Thiamine hydrochloride	Sangon Biotech	A500986-0050
L-Lysine	Sangon Biotech	A602759-0025
L-Arginine	Sangon Biotech	A600205-0100
L-Alanine	Sangon Biotech	A413248-0500
L-Asparagine	Sangon Biotech	A422874-0100
L-Aspartic acid	Sangon Biotech	A600091-0250
L-Cysteine	Sangon Biotech	A600132-0100
L-Glutamine	Sangon Biotech	A430713-0050
L-Glutamic acid	Sangon Biotech	A600221-0500
Glycine	Sangon Biotech	A610236-0500
L-Histidine	Sangon Biotech	A604351-0050
L-Isoleucine	Sangon Biotech	A600914-0050
L-Leucine	Sangon Biotech	A600922-0100
L-Methionine	Sangon Biotech	A610346-0025
L-Phenylalanine	Sangon Biotech	A600991-0025
L-Proline	Sangon Biotech	A600923-0100
L-Serine	Sangon Biotech	A422880-0500
L-Threonine	Sangon Biotech	A610919-0100

L-Tryptophan	Sangon Biotech	A601911-0050
L-Tyrosine	Sangon Biotech	A601932-0100
L-Valine	Sangon Biotech	A600172-0025
Lysogeny Broth	Sangon Biotech	A507002-0250
Agar	Sangon Biotech	A505255-0250
Kanamycin	Sangon Biotech	A600286-0025

Reference

1. Livak, K.J. and T.D. Schmittgen. Analysis of Relative Gene Expression Data Using Real-Time Quantitative PCR and the 2−ΔΔCT Method. Methods. 2001; **25**(4): 402-408.
2. Jiang, Y., B. Chen, C. Duan, B. Sun, J. Yang, and S. Yang. Multigene Editing in the Escherichia coli Genome via the CRISPR-Cas9 System. Applied and Environmental Microbiology. 2015; 81(7): 2506-2514.
